# Is there an association between intravenous immunoglobulin resistance and coronary artery lesion in Kawasaki disease?—Current evidence based on a meta-analysis

**DOI:** 10.1371/journal.pone.0248812

**Published:** 2021-03-25

**Authors:** Xiaolan Zheng, Jinhui Li, Peng Yue, Lei Liu, Jiawen Li, Kaiyu Zhou, Yimin Hua, Yifei Li

**Affiliations:** 1 Department of Pediatrics in West China Second University Hospital, Sichuan University, Chengdu, Sichuan, China; 2 Key Laboratory of Birth Defects and Related Diseases of Women and Children of MOE, West China Second University Hospital, Sichuan University, Chengdu, Sichuan, China; Mansoura University, EGYPT

## Abstract

**Background:**

Coronary artery lesion (CAL) caused by Kawasaki disease (KD) is a leading cause of acquired heart disease in children. Initial treatment of intravenous immunoglobulin (IVIG) can reduce the incidence of CAL. Although most of the current studies have shown a certain correlation between CAL and IVIG resistance, the conclusions are not completely consistent. Thus, we performed this meta-analysis to evaluate the association between IVIG resistance and CAL in KD.

**Methods:**

PubMed, EMBASE, the Cochrane Central Register of Controlled Trials, and China National Knowledge Infrastructure through April 21, 2020 were searched to detect relevant studies. Data analysis was performed with STATA 15.1.

**Results:**

A total of 53 relevant studies were eligible to this analysis, including 30312 KD patients, of which 4750 were IVIG resistance and 25562 were responders. There was a significant difference found between IVIG resistance and IVIG response groups in the incidence of CAL (P < 0.001, odds ratio (OR), 3.89; 95% confidence interval (CI) (3.18, 4.75)). The heterogeneity test results showed that the I^2^ value was 74.8%. The meta-regression analysis showed that the study regions might be the sources of heterogeneity. The subgroup analysis suggested that the incidence of CAL in the IVIG resistance group was still higher than that in the IVIG response group under different regions, IVIG resistance diagnostic criteria, CAL diagnostic criteria, and study types. Meanwhile, the sensitivity analysis did not find any significant impact from every single study.

**Conclusions:**

This is the first meta-analysis to reveal the incidence of CAL was associated with IVIG resistance in KD patients. Further well-designed studies with uniform criteria are needed to evaluate the incidence of CAL in IVIG resistant patients.

## Introduction

Kawasaki disease (KD) is an acute vasculitis of unknown etiology that predominantly affects children, first identified in Japan and now reported worldwide [1]. KD may cause coronary artery lesion (CAL) and is currently the leading cause of acquired heart disease in children in developed countries [2]. The American Heart Association (AHA) recommends the standard treatment regimen for the acute phase of KD involves administering intravenous immunoglobulin (IVIG) 2 g·kg-1 and aspirin [3]. Previous studies have indicated that the incidence of CAL is highly correlated with dose and infusion timing of IVIG, not aspirin [4, 5]. Early use of IVIG in KD can effectively reduce the incidence of CAL from 25% to about 4% [3]. However, up to 20% of KD patients may fail to respond to IVIG [6]. Even though the precise molecular mechanism of CAL and IVIG in KD are still unclear, many clinical studies suggest IVIG resistance has deeply associated with the occurrence of CAL [7–9]. Thus, a great deal of literature have used this as a starting point to study the indicators for predicting IVIG resistance [10–13], with the expectation that additional therapeutic measures can be taken to reduce the incidence of CAL through early diagnosis of IVIG resistance.

Although most of the current studies have shown the association between CAL and IVIG resistance, the conclusions are not completely consistent. Besides, there is still a lack of comprehensive and systematic analysis of this issue. Therefore, we performed this meta-analysis for the first time to evaluate the association between IVIG resistance and CAL in KD.

## Materials and methods

### Study protocol

We generated this meta-analysis followed a predetermined protocol by the recommendations of the Preferred Reporting Items for Systematic Reviews and Meta-Analyses (PRISMA) Statement [14]. The PRISMA checklist could be found in S1 Table. This study was registered with PROSPERO (CRD42020181359).

### Search strategy

We searched multiple databases, including PubMed, EMBASE, the Cochrane Central Register of Controlled Trials (CCTR), and China National Knowledge Infrastructure (CNKI) through April 21, 2020 to identify relevant studies. We searched the PubMed as follows: (((Mucocutaneous Lymph Node Syndrome [MeSH Terms] OR Kawasaki disease OR Kawasaki syndrome) AND (Immunoglobulins, Intravenous [MeSH Terms] OR IVIG OR Intravenous Immune Globulin OR Immune Globulin, Intravenous OR Intravenous Immunoglobulins)) AND (((resistance) OR (resistant)) OR (nonresponse) OR (refractory))) AND (coronary artery). Search terms and corresponding results of EMBASE, CCTR, and CNKI are shown in S1 Appendix. The language was limited to English.

### Study selection

We initially screened researches by the title and abstract. A full-text search that retrieved potentially relevant reports was assessed for compliance using the inclusion and exclusion criteria. Inter-rater reliability for the study selection was calculated using the kappa statistic [15]. Studies that meet all the following criteria were included: 1) All cases were KD patients; 2) non-randomized or randomized controlled clinical trials or cohort studies evaluating the incidence of CAL in KD patients, including IVIG resistance and IVIG response. Meanwhile, researches meeting any of the following criteria were excluded: 1) Initial IVIG treatment is not the AHA recommended standard dose (2 g·kg^-1^); 2) conference articles, reviews, or abstracts; 3) The sample size included in the study was too small (n < 60); 4) duplicate reports.

### Data collection and assessment of study quality

Two investigators (Xiaolan Zheng, Jinhui Li) independently assessed the eligibility of studies by the title and abstract, and a third reviewer (Peng Yue) determining divergence based on the inclusion or exclusion criteria and the quality of literature, and consultation with a fourth reviewer (Yifei Li) if necessary. Besides, we used the Newcastle-Ottawa Scale (NOS) to assess the quality of the included researches [16]. Additionally, literature with a score of fewer than five stars was considered as of low quality and needed to be excluded.

### Data synthesis and analysis

We calculated the odds ratio (OR) with 95% confidence intervals (CI) for each outcome of interest then pooled them into an independent meta-analysis. KD children with IVIG resistance were the experimental group, and the control group was the IVIG responders. The outcome was the incidence of CAL in the experimental group or the control group, including coronary artery aneurysm (CAA), coronary artery dilatation (CAD) or ectasia, which was defined according to the diagnostic criteria of the American Heart Association (AHA) Z-score [3] or the Japan Kawasaki Disease Research Committee (JKDRC) [17].

### Publication bias

We used Egger’s regression by STATA software version 15.1 to test the publication bias of the include studies. Each study is represented by a dot, and the asymmetrical distribution of dots between the regression line suggested publication bias, where the quantified result showed that P < 0.05 [18]. When publication bias occurs, we use the trim-and-fill method further to evaluate the impact of publication bias on the results. If there is no significant difference between the results before and after the test of the trim-and-fill method, it indicates that the results are stable and reliable even if there is publication bias [19].

### Heterogeneity

Heterogeneity was investigated by the Chi-squared test and was considered as statistically significant when P < 0.05 in these tests. The I^2^ values can range from 0 to 100%, higher than 25%, 50%, and 75%, suggesting low, moderate, and high heterogeneity, respectively [20]. When the I^2^ value exceeded 50%, we used the random-effects (M-H method) for analysis; otherwise, the fixed-effects (M-H method) was used.

### Meta-regression and subgroup analysis

The meta-regression was conducted to investigate the potential factor of leaded heterogeneity. The subgroups analysis was performed according to the study regions, IVIG resistance diagnostic criteria, CAL diagnostic criteria, and study types.

### Sensitivity analysis

Sensitivity analysis was conducted for every study to investigate whether any single study caused undue weight in the results of the meta-analysis.

### Statistical analysis

Data analysis was performed with STATA 15.1. The numbers of children who developed CAL in each study were converted to OR. Meanwhile, heterogeneity, publication bias, and sensitivity analysis were also conducted by STATA 15.1.

## Results

### Search results

**Fig 1:**
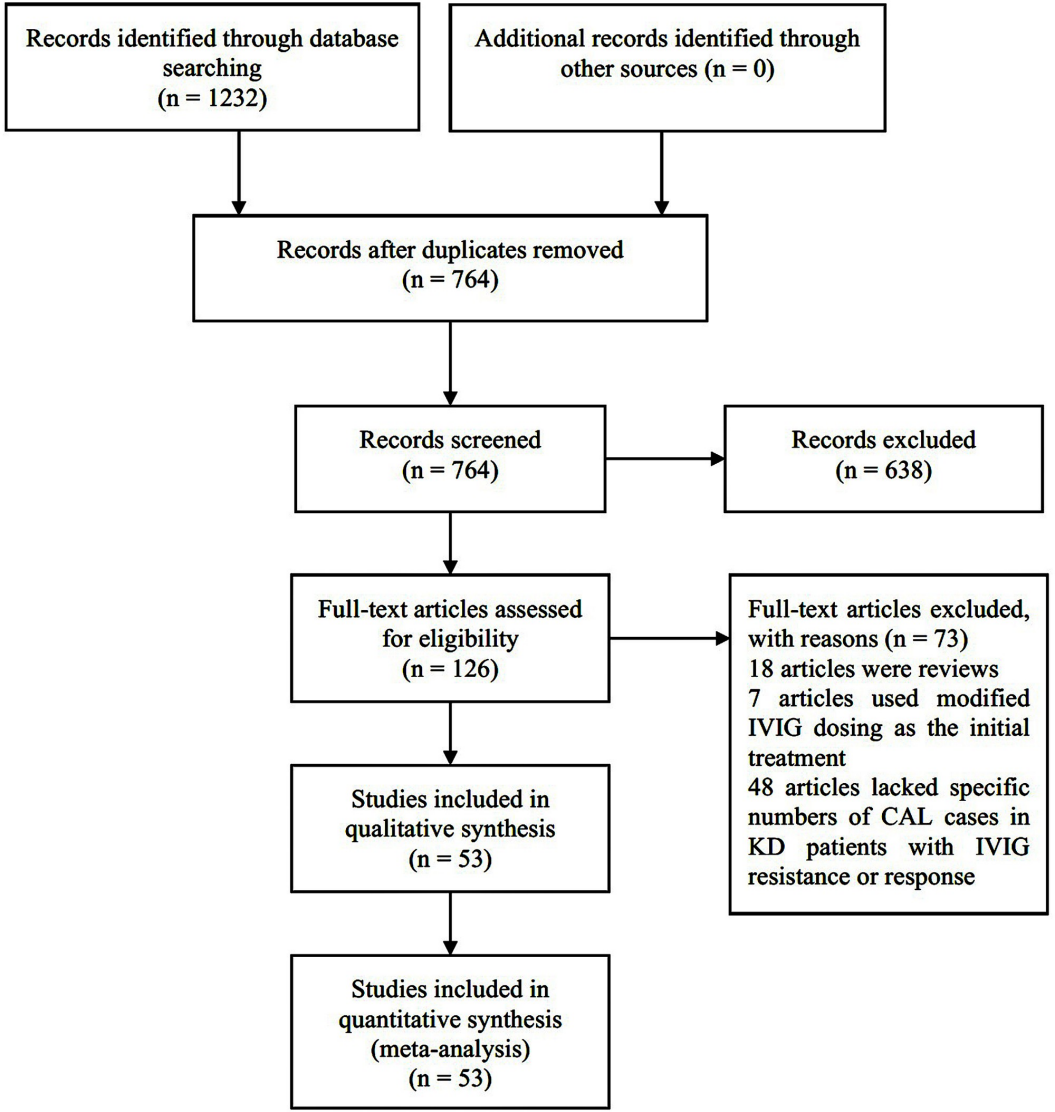
Flowchart of the study selection process.

Initially, 1232 potentially relevant studies were retrieved by the search method, of which 126 were considered as of interest by titles and abstracts. However, 73 studies were excluded by reading the complete researches due to article type (n = 18), using the modified IVIG dosing as the initial treatment (n = 7), and lacking specific numbers of CAL cases in KD patients with IVIG resistance or response (n = 48). Ultimately, 53 articles [8, 10, 11, 13, 21–69] were included in the meta-analysis. The kappa test showed the kappa value of agreement during the systematic searches was 0.86. The research selection process was shown in Fig 1.

### Study characteristics

A total of 30312 KD patients were included, of which 4750 were IVIG resistance, and 25562 were responders. The 53 studies included were from 11 countries in three regions (Asia, North America, and Europe). Besides, the criteria for the diagnosis of KD resistance was not consistent, among them, IVIG resistance of six studies was defined as persistent fever ≥ 24h after the end of the first IVIG infusion, 21 studies to define the time for ≥ 36h, or otherwise, 17 studies was defined as ≥ 48h, and for the remaining nine studies was defined as a second dose of IVIG for persistent or recrudescent fever without mentioning the specific time. Moreover, the CAL diagnostic criteria in all the included literatures were not consistent, among which, JKDRC criteria was used in 29 studies, AHA criteria was used in 14 studies, JKDRC or AHA criteria was both used in eight studies, and the remaining two studies did not report which criteria was used. All included studies were non-randomized trials, including 5 prospective studies and 48 retrospective studies. Moreover, all the included reports were awarded ≥ six stars and qualified as high quality. The characteristics of KD children in all involved studies were illustrated in Table 1.

**Table 1:** Characteristics of studies in meta-analysis.

No.	First author	Year	Countries	Study type	Diagnostic criteria of CAL	Diagnostic time of IVIG resistance	Age (years) (IVIG resistance/IVIG response)	Male (%) (IVIG resistance/IVIG response)	KD patients (IVIG resistance/IVIG response)	CAL patients (IVIG resistance/IVIG response)	Follow-up (weeks)	NOS
1	Hashino	2001	Japan	retrospective	JKDRC	48h	N/R	N/R	35/227	9/0	N/R	6
2	Hsieh	2004	China	retrospective	JKDRC	72h	2.8/1.7	56.5/61.5	9/153	3/20	8	7
3	Sittiwangkul	2006	Thailand	retrospective	JKDRC/AHA	36h	1.4/1.6	44.0/51.0	9/61	7/10	52	6
4	Sano	2006	Japan	retrospective	AHA	24h	2.2/2.3	N/R	22/90	17/9	> 52	6
5	Furukawa	2007	Japan	retrospective	JKDRC	36h	N/R	54.0/60.1	63/348	7/1	N/R	7
6	Uehara	2008	Japan	retrospective	JKDRC	N/R	2.0/1.9	N/R	1286/5044	333/402	N/R	6
7	Tremoulet	2008	USA	retrospective	AHA	48h	2.0/2.4	63.3/60.9	60/302	29/109	4	8
8	Sabharwal	2009	Canada	retrospective	JKDRC	36h	N/R	N/R	181/1117	54/204	6–8	6
9	Mamtani	2010	Japan	prospective	JKDRC	36h	N/R	N/R	25/68	18/5	N/R	6
10	Weng	2010	China	retrospective	JKDRC	48h	2.7/2.0	50.0/57.4	20/136	12/44	8	7
11	Do	2010	Korea	retrospective	JKDRC	48h	2.7/2.5	61.5/66.7	13/64	5/7	52	7
12	Kuo	2010	China	retrospective	JKDRC	48h	1.6/1.6	N/R	20/111	6/24	4	7
13	Hwang	2011	Korea	retrospective	JKDRC	24h	2.8/2.4	60.8/54.4	23/206	14/37	4	6
14	Sittiwangkul	2011	Thailand	retrospective	JKDRC	N/R	N/R	N/R	18/137	11/29	52	6
15	Iwashima	2011	Japan	retrospective	JKDRC	48h	N/R	N/R	108/325	30/23	N/R	7
16	Kim	2011	Korea	prospective	JKDRC	48h	3.0/2.4	68.2/53.3	22/107	7/3	8	6
17	Yoshimura	2013	Japan	retrospective	AHA	24h	1.0/2.0	47.0/73.0	17/63	8/11	4	6
18	Ou-Yang	2013	China	retrospective	JKDRC	48-72h	1.0/1.8	40.0/63.8	5/58	5/7	12	7
19	Park	2013	Korea	retrospective	AHA	36h	2.3/2.2	63.3/50.9	30/279	13/19	N/R	7
20	Teraguchi	2013	Japan	prospective	JKDRC	36h	N/R	58.5/62.2	41/196	12/5	N/R	6
21	Cho	2014	Korea	retrospective	AHA	36h	N/R	52.9/55.6	17/135	8/29	10–62	6
22	Yi	2014	Korea	retrospective	JKDRC	36h	N/R	N/R	17/47	7/17	> 4	7
23	Adjagba	2014	Canada	prospective	AHA	36h	N/R	N/R	16/93	6/16	12	6
24	Tajima	2015	Japan	retrospective	JKDRC	N/R	1.7/2.3	71.0/66.7	31/60	10/3	4	8
25	Loomba	2015	USA	retrospective	AHA	N/R	3.5/3.5	69/52	58/124	4/4	6–8	7
26	Ha	2015	Korea	retrospective	JKDRC	48h	2.9/2.8	60.4/56.2	222/365	36/26	10	8
27	Maggio	2016	Italy	retrospective	AHA	N/R	2.2/2.3	50.0/51.8	14/50	4/11	52	6
28	Lee	2016	Korea	retrospective	JKDRC/AHA	36h	3.0/2.4	55.9/55.3	34/253	21/9	N/R	7
29	Xu	2016	China	retrospective	JKDRC	N/R	N/R	N/R	44/378	18/65	12	6
30	Okuma	2016	Japan	retrospective	JKDRC	N/R	1.75/2	45.5/48	22/89	5/3	N/R	8
31	Kawamura	2016	Japan	retrospective	JKDRC	24h	2.8/2.1	57.6/57.8	85/320	9/1	N/R	7
32	Xie	2017	China	retrospective	JKDRC	48h	N/R	N/R	56/504	27/126	4	6
33	Berdej-Szczot	2017	Poland	retrospective	N/R	36h	N/R	N/R	8/65	1/12	26	6
34	Kimura	2017	Japan	retrospective	JKDRC	48h	N/R	N/R	147/476	12/1	N/R	7
35	Chbeir	2018	France	retrospective	JKDRC/AHA	48h	N/R	53.3/60.7	45/112	14/17	52	8
36	Hua	2018	China	retrospective	JKDRC/AHA	48h	N/R	N/R	380/1735	118/371	N/R	6
37	Kim	2018	Korea	retrospective	JKDRC	N/R	2.8/2.7	60.5/52.2	524/4627	90/435	N/R	7
38	Miyakoshi	2018	Japan	retrospective	JKDRC	24h	2.5/1.8	70.0/58.0	98/224	17/15	N/R	8
39	Ahn	2018	Korea	prospective	JKDRC/AHA	36h	N/R	N/R	38/227	14/31	52	6
40	Fabi	2018	Italy	retrospective	JKDRC/AHA	36h	2.6/2.8	69.8/55.6	43/214	16/42	N/R	6
41	Bar-Meir	2018	Israel	retrospective	AHA	36h	2.4/2.7	62.0/65.0	42/270	20/67	4	7
42	Clark	2018	USA	retrospective	AHA	36h	2.6/2.8	67.2/64.6	67/325	29/77	104	8
43	Chantasiriwan	2018	Thailand	retrospective	AHA	36h	1.4/1.4	85.0/62.0	26/191	9/46	8	8
44	Gámez-González	2018	Japan	retrospective	N/R	48h	2.7/2.6	69.3/54.7	101/318	10/1	N/R	6
45	Tan	2019	China	retrospective	JKDRC	48h	2.5/2.7	66.7/61.6	260/3745	177/1733	N/R	7
46	Kong	2019	China	retrospective	JKDRC/AHA	36h	2.1/2	58.7/60.8	29/271	7/41	6–8	8
47	Fernandez-Cooke	2019	Spanish	retrospective	AHA	36h	N/R	N/R	98/527	15/45	26	7
48	Wang	2019	China	retrospective	AHA	36h	2.2/2.2	50/54	50/50	12/3	N/R	6
49	Yılmazer	2019	Turkey	retrospective	AHA	36h	2.95/2.75	61.1/62.7	18/102	8/27	52	7
50	Amano	2019	Japan	retrospective	JKDRC	24h	2.63/3.01	69.7/56.5	33/42	17/9	N/R	6
51	Liu	2020	China	retrospective	JKDRC/AHA	36h	2.8/2.9	36.4/56.5	11/131	2/6	N/R	7
52	Piram	2020	France	retrospective	JKDRC	N/R	N/R	59.8/55.7	92/323	56/105	12	7
53	Türkuçar	2020	Turkey	retrospective	JKDRC	48h	2.83/3	58.7/58.3	17/77	8/17	2	7

CAL = coronary artery lesion, IVIG = intravenous immunoglobulin, KD = Kawasaki Disease, NOS = Newcastle-Ottawa Scale, JKDRC = Japan Kawasaki Disease Research Committee, AHA = American Heart Association, N/R = not report.

### Data synthesis and analysis

**Fig 2:**
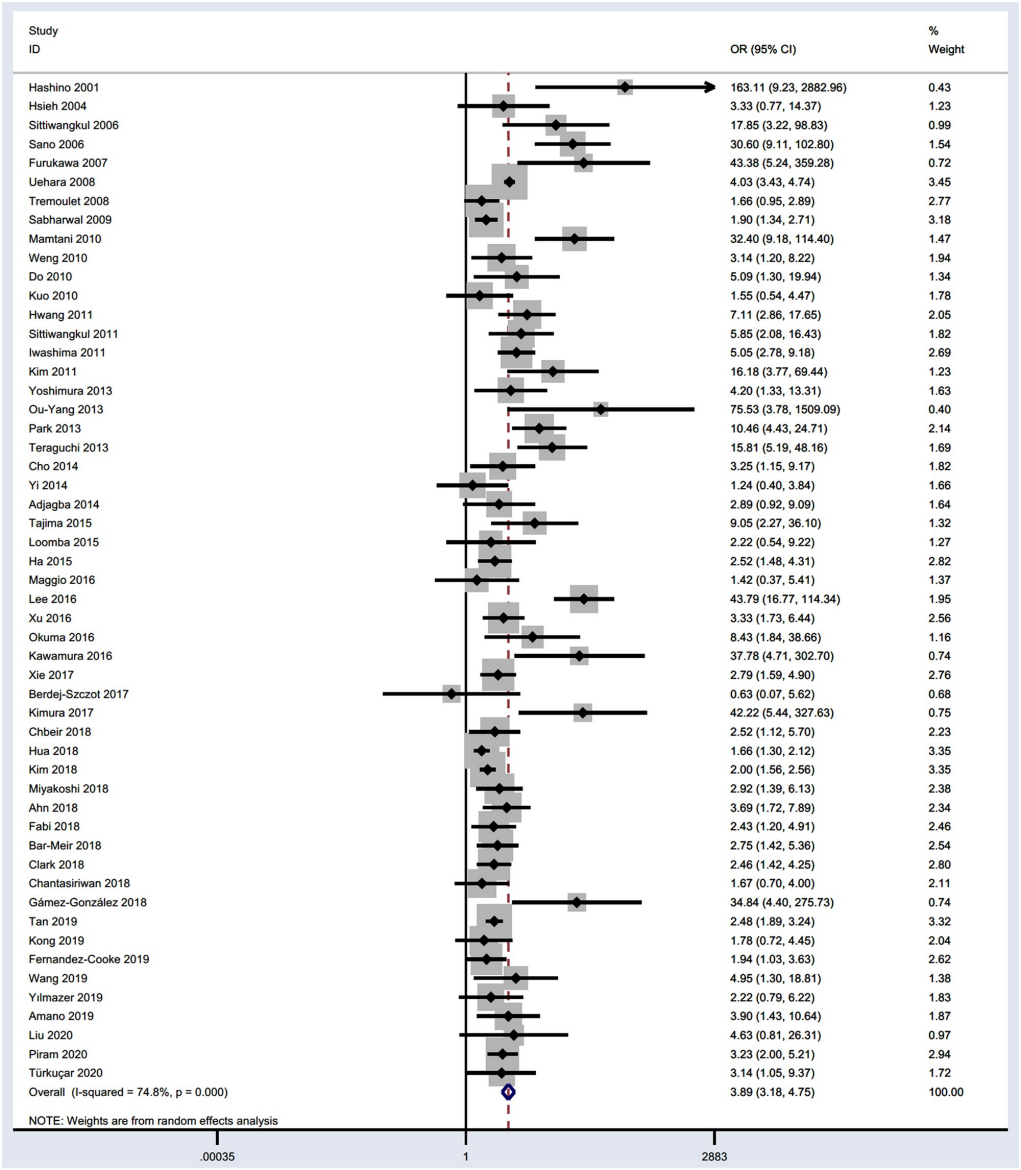
Forest plot for the CAL of IVIG resistant children vs IVIG responders. OR, odds ratio. CI, confidence interval.

The OR value and 95%CI of the CAL cases of IVIG resistant children in each study were calculated by STATA 15.1, and all the results were pooled into an independent meta-analysis. The results showed a significant difference between IVIG resistance and IVIG response groups in the incidence of CAL (P < 0.001, OR, 3.89; 95%CI (3.18, 4.75)) (Fig 2). Due to the I^2^ value was 74.8%, we used the random-effects (M-H method) for analysis.

### Publication bias

The result of the Egger’s regression test was shown an asymmetric distribution indicated that publication bias existed (P = 0.002, t = 3.19, 95%CI (0.47, 2.07)) (S1A Fig). Then, we use the trim-and-fill method further to evaluate the impact of publication bias on the results. The funnel plot of publication bias used the trim-and-fill method showed that no significant difference between the results before (P <0.001, Z = 13.26, 95%CI (1.16, 1.56)) and after filled 13 studies (P <0.001, Z = 9.04, 95%CI (0.80, 1.25)) (S1B Fig), suggesting that the results of meta-analysis were robust even with publication bias.

### Meta-regression and subgroup analysis

**Fig 3:**
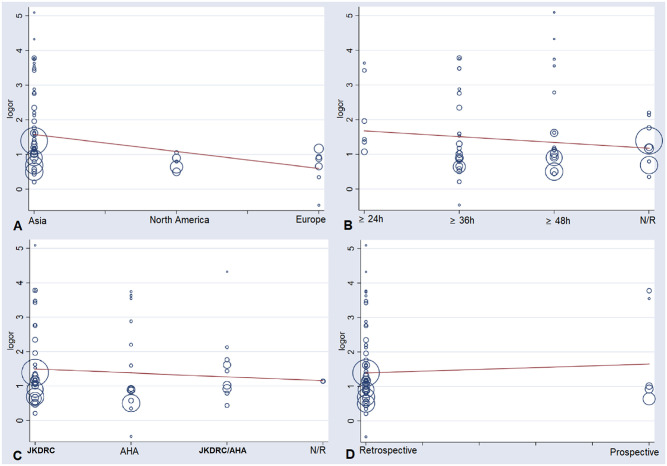
The meta-regression of the enrolled studies. (A) the study regions might the sources of heterogeneity (P = 0.012, t = -2.62, 95%CI (0.42, 0.89)). (B) IVIG resistance diagnostic criteria were not the impact factor on the heterogeneity (P = 0.269, t = -1.12, 95%CI (0.63, 1.14)). (C) CAL diagnostic criteria could not impact the homogeneity too (P = 0.473, t = -0.72, 95%CI (0.65, 1.22)). (D) the study type was not a dramatic impact factor on the homogeneity (P = 0.571, t = 0.57, 95%CI (0.52, 3.18)). OR, odds ratio. CI, confidence interval.

As the results of meta-analysis showed moderate to high heterogeneity, we used the meta-regression to investigate the origins of heterogeneities. According to the results (Fig 3), the study regions might be the sources of heterogeneity (P = 0.012, t = -2.62, 95%CI (0.42, 0.89)) (Fig 3A). Besides, IVIG resistance diagnostic criteria were not the impact factor on the heterogeneity (P = 0.269, t = -1.12, 95%CI (0.63, 1.14)) (Fig 3B). Meanwhile, CAL diagnostic criteria could not impact the homogeneity too (P = 0.473, t = -0.72, 95%CI (0.65, 1.22)) (Fig 3C). Additionally, the meta-regression also suggested the study type was not a dramatic impact factor (P = 0.571, t = 0.57, 95%CI (0.52, 3.18)) (Fig 3D). The subgroup analysis suggested that the incidence of CAL in the IVIG resistance group was still higher than that in the IVIG response group under different regions (OR(95%CI): Asia, 4.80(3.76, 6.13); North America, 2.00(1.56, 2.57); Europe, 2.46(1.82, 3.32)) (S2 Fig), IVIG resistance diagnostic criteria(OR(95%CI): ≥ 24h, 7.08(3.31, 15.15); ≥ 36h, 4.11(2.72, 6.20); ≥ 48h, 3.40(2.42, 4.76); N/R, 3.34(2.34, 4.77)) (S3 Fig), CAL diagnostic criteria (OR(95%CI): JKDRC, 4.29(3.32, 5.56); AHA, 3.09(2.11, 4.52); JKDRC/AHA, 3.99(2.11, 7.56); N/R, 4.76(0.09, 245.28)) (S4 Fig), and study types (OR(95%CI): retrospective, 3.75(3.06, 4.59); prospective, 5.77(2.16, 15.42)) (S5 Fig).

### Sensitivity analysis

**Fig 4:**
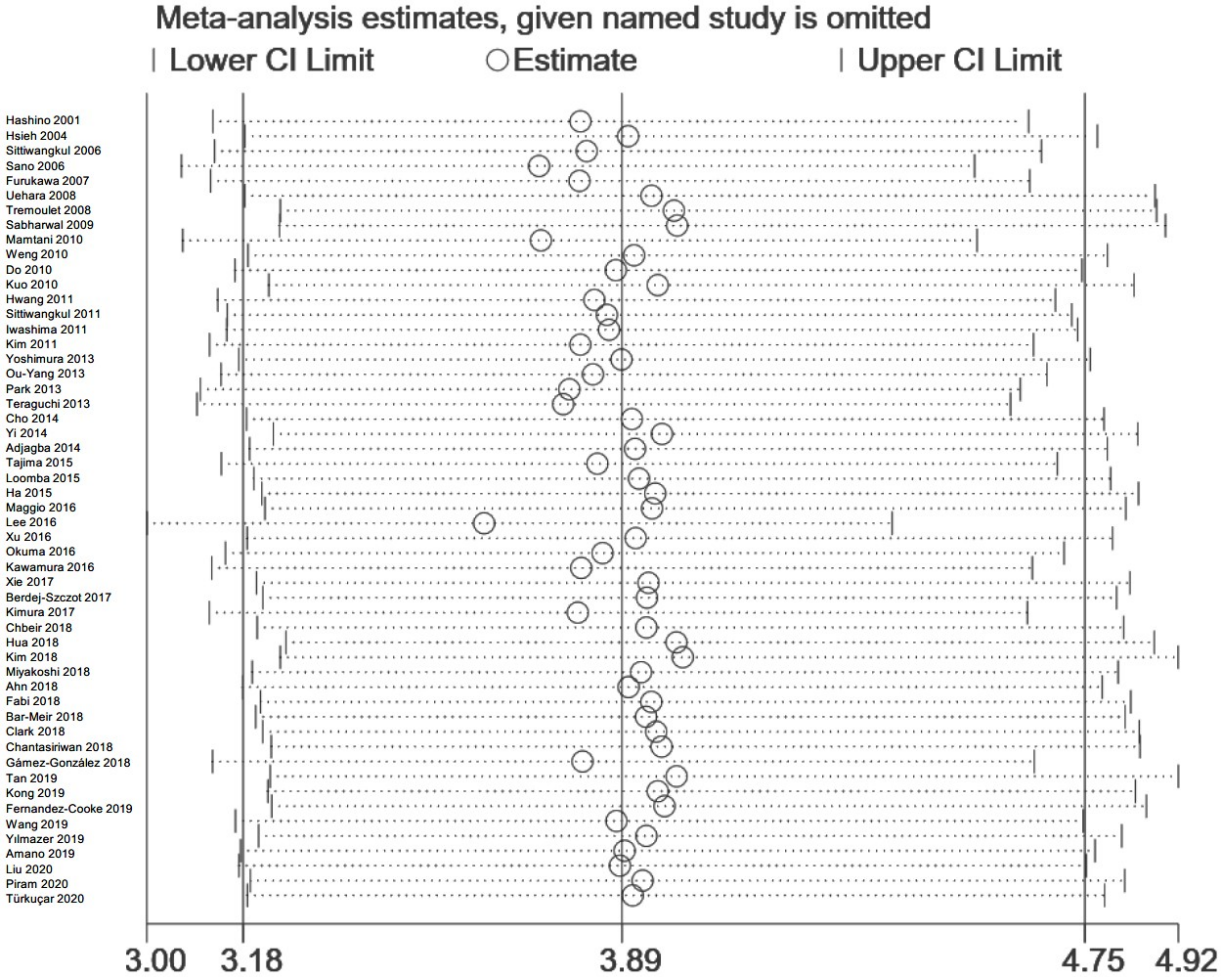
Sensitivity analysis of the results.

Sensitivity analysis was performed by STATA 15.1, and we did not detect any significant impact from every single research in the result (Fig 4).

## Discussion

CAL caused by KD is a significant component of acquired heart disease in children in many countries [70, 71]. Although initial IVIG treatment can effectively reduce the incidence of CAL, IVIG resistance still exists in some children. This is the first meta-analysis to evaluate the association between IVIG resistance and CAL in KD. A total of 53 relevant studies were involved in our meta-analysis, including 30312 KD patients (4750 IVIG resistant patients and 25562 responders). The results showed a significant difference between IVIG resistance and IVIG response groups in the incidence of CAL in KD patients.

CAL is the major complications to KD, which reveals a poor prognosis. So that, hundreds of researchers had been involved in the studies of risk factors related with CAL in the last decades. IVIG resistance is one of the dominant adverse effects during acute phase treatment, which brought into observations. Even several previous studies [7–9] have suggested that the incidence of CAL was significantly higher in IVIG resistant populations. However, this issue still be debating as a series of researches demonstrated the negative association between IVIG resistance and CAL. So that it is important to draw a clear relationship of IVIG administration effects and CAL based on this meta-analysis. To predict IVIG resistance, a variety of scoring systems have been designed, and Kobayashi and Egami are the most popular ones [72, 73]. Additionally, a lot of researches have been conducted to identify risk factors for IVIG resistant KD [74–78]. Patients’ ages, biomarkers including CRP, ESR, total bilirubin, AST, ALT, and Pro-BNP were identified positively correlation with IVIG resistance. Unfortunately, the sensitivity and specificity of such indicators are not qualified to be applied alone. So that an integrative scoring system need to be developed to predict IVIG resistance. And the different genetic backgrounds were also considered to be involved in various clinical results. Therefore, our study was intended to conduct subgroup analysis on ethical race. Due to the included literature was mainly divided by region and lacked ethnic information, this paper finally adopted region for subgroup analysis. In addition, some studies have shown that children with younger or elder age are more likely to demonstrate a drug resistance. However, since the included literature in this paper also lacks the segmentation of the age of the included children, further subgroup analysis at different ages cannot be carried out. These questions need to be analyzed in more well-designed studies in the future.

It is worth noting that in our literature search process, we found that majority of studies indicated that IVIG resistance might lead to an increase in the incidence of CAL, while there were few studies with different opinions. In this case, we should be aware of the possibility of publication bias. And then, the Egger’s test turned out publication bias existed. Next, we use the trim-and-fill method to evaluate the impact of publication bias on the results. Finally, the trim-and-fill method suggested the results were relatively robust even with publication bias.

As the heterogeneity test results showed that the I^2^ value was 74.8%, we conducted the meta-regression analysis to investigate the origins of heterogeneities. The results showed that the study regions might be the sources of heterogeneity. The subgroup analysis suggested that the incidence of CAL in the IVIG resistance group was still higher than that in the IVIG response group under different regions, IVIG resistance diagnostic criteria, CAL diagnostic criteria, and study types. These results indicate that the IVIG resistance group has a higher risk of CAL than the IVIG response group, even under a variety of conditions. Since IVIG was first used in Kawasaki disease in 1982 [79], CAL has decreased significantly. Gradually, the occurrence of IVIG resistance has attracted extensive attention. Research on the prediction and treatment of IVIG resistance has been a research hotspot for many years. Nevertheless, the mechanism by which IVIG reduces the incidence of CAL and IVIG resistance is not yet clear, and the mechanism by which CAL is also unclear, probably because the etiology of KD is still unknown.

Current follow up drug therapy for KD children with IVIG resistance mainly includes the second dose of IVIG, methylprednisolone (MP), infliximab, interleukin 1 receptor antagonist or cyclosporin [3]. However, KD patients who fail to respond to 2 doses of IVIG present a unique challenge [80]. A meta-analysis comprising 372 refractory KD patients conducted in 2019 by Chan et al. [81] revealed that infliximab, MP, and second IVIG infusion showed no significant differences in the cardioprotective effect or the rate of treatment resistance. So far, there is still no accepted treatment algorithm for refractory KD patients [82]. Among the 53 literatures included in our study, there were few literatures that provided detailed adjunct therapies and outcomes for IVIG resistance, so we could not perform analysis to evaluate the effect of different treatment regimens on the incidence of CAL in IVIG resistance. A meta-analysis [83] focused on this issue showed that steroids were more effective at reducing fever than second dose of IVIG, but was no difference in the incidence of CAL. It should be noted that the current AHA guidelines do not recommend the routine use of steroids [3]. Besides, studies which comparing infliximab with the second dose of IVIG in IVIG resistance indicated that infliximab reduced fever duration, but the outcome of coronary was similar [84, 85]. In general, there have been no robust clinical trials comparing second-line regiments for IVIG resistance in KD [86], further research into adjunct therapies is an area for future work.

Our study has several limitations. First, most studies lacked information about the adjunct therapies of IVIG resistance. Although previous studies suggested that adjuvant therapy had no significant effect on the incidence of CAL in IVIG resistance, it may still cause bias to the results of this study. Second, the follow-up time of many studies was not reported, which may also lead bias to the results. Third, most of the included studies were retrospective trials, which could lead to biased results. Therefore, further well-designed studies with uniform criteria are needed to evaluate the incidence of CAL in IVIG resistant patients and IVIG responders.

## Conclusions

This is the first meta-analysis to reveal the incidence of CAL was associated with IVIG resistance in KD patients. Further well-designed studies with uniform criteria are needed to evaluate the incidence of CAL in IVIG resistant patients.

## Supporting information

S1 Table PRISMA checklist. (DOC)

S1 Appendix Search strategies for EMBASE, CCRT, and CNKI. (DOCX)

S1 Fig The assessment results of potential publication bias. (A) Egger’s publication bias plots for the assessment of potential publication bias. Asymmetry of the dot distribution between regression lines showed potential publication bias, P = 0.002, t = 3.19, 95%CI (0.47, 2.07). (B) The funnel plot of publication bias by the trim-and-fill method. After filled 13 potentially missing studies, the funnel plots were symmetrical. CI, confidence interval.
(PDF)

S2 Fig The results of subgroup analysis by study regions. (PDF)

S3 Fig The results of subgroup analysis by IVIG resistance diagnostic criteria. (PDF)

S4 Fig The results of subgroup analysis by CAL diagnostic criteria. (PDF)

S5 Fig The results of subgroup analysis by study types. (PDF)
