# Supplementary material for: Is there an association between intravenous immunoglobulin resistance and coronary artery lesion in Kawasaki disease?—Current evidence based on a meta-analysis
Source: PLoS One. 2021 Mar 25;16(3):e0248812. doi: 10.1371/journal.pone.0248812 (PMC7993784; doi:10.1371/journal.pone.0248812)
Supplement: S1 Appendix — (DOCX) [file pone.0248812.s002.docx]

Search strategies for EMBASE, the Cochrane Central Register of Controlled Trials, and China National Knowledge Infrastructure.

A: Embase database was searched as follows: #1'mucocutaneous lymph node syndrome'/exp OR 'mucocutaneous lymph node syndrome' OR 'kawasaki disease'/exp OR 'kawasaki disease' OR 'kawasaki syndrome'/exp OR 'kawasaki syndrome', #2 'immunoglobulin'/exp OR 'immunoglobulin' OR 'IVIG'/exp OR 'IVIG', #3 'resistance'/exp OR resistance OR resistant OR nonresponse OR refractory, #4 coronary AND ('artery'/exp OR artery) . #1 AND #2 AND #3 AND #4

| Embase | | |
| --- | --- | --- |
| 1 | 'mucocutaneous lymph node syndrome'/exp OR 'mucocutaneous lymph node syndrome' OR 'kawasaki disease'/exp OR 'kawasaki disease' OR 'kawasaki syndrome'/exp OR 'kawasaki syndrome' | 11,768 |
| 2 | 'immunoglobulin'/exp OR 'immunoglobulin' OR 'IVIG'/exp OR 'IVIG' | 743,533 |
| 3 | 'resistance'/exp OR resistance OR resistant OR nonresponse OR refractory | 1,695,631 |
| 4 | coronary AND ('artery'/exp OR artery) | 517,508 |
| 5 | #1 AND #2 AND #3 AND #4 | 665 |

## B: the Cochrane Central Register of Controlled Trials database was searched as follows: (‘mucocutaneous lymph node syndrome’ OR ‘Kawasaki disease’ OR ‘Kawasaki syndrome’):ti,ab,kw AND (‘immunoglobulin’ OR ‘Intravenous Immune Globulin’ OR ‘IVIG’ OR ‘Intravenous Immunoglobulins’):ti,ab,kw AND (‘resistance’ OR ‘resistant’ OR ‘nonresponse’ OR ‘refractory’):ti,ab,kw AND (‘coronary artery’):ti,ab,kw

| the Cochrane Central Register of Controlled Trials | | |
| --- | --- | --- |
| 1 | (‘mucocutaneous lymph node syndrome’ OR ‘Kawasaki disease’ OR ‘Kawasaki syndrome’):ti,ab,kw | 290 |
| 2 | (‘immunoglobulin’ OR ‘Intravenous Immune Globulin’ OR ‘IVIG’ OR ‘Intravenous Immunoglobulins’):ti,ab,kw | 13341 |
| 3 | (‘resistance’ OR ‘resistant’ OR ‘nonresponse’ OR ‘refractory’):ti,ab,kw | 85680 |
| 4 | (‘coronary artery’):ti,ab,kw | 33754 |
| 5 | #1 AND #2 AND #3 AND #4 | 48 |

## C: China National Knowledge Infrastructure database was searched as follows:  (mucocutaneous lymph node syndrome OR Kawasaki disease OR Kawasaki syndrome) AND (immunoglobulin OR IVIG OR Intravenous Immunoglobulins) AND (resistance OR resistant OR nonresponse OR refractory) AND (coronary artery) in Title Abstract Keyword.

| China National Knowledge Infrastructure database | | |
| --- | --- | --- |
| 1 | (mucocutaneous lymph node syndrome OR Kawasaki disease OR Kawasaki syndrome) in Title Abstract Keyword | 13559 |
| 2 | (immunoglobulin OR IVIG OR Intravenous Immunoglobulins) in Title Abstract Keyword | 33276 |
| 3 | (resistance OR resistant OR nonresponse OR refractory) in Title Abstract Keyword | 85680 |
| 4 | (coronary artery) in Title Abstract Keyword | 337 |
| 5 | #1 AND #2 AND #3 AND #4 | 251 |
