# Supplementary figures and images for: Is there an association between intravenous immunoglobulin resistance and coronary artery lesion in Kawasaki disease?—Current evidence based on a meta-analysis
Source: PLoS One. 2021 Mar 25;16(3):e0248812. doi: 10.1371/journal.pone.0248812 (PMC7993784; doi:10.1371/journal.pone.0248812)

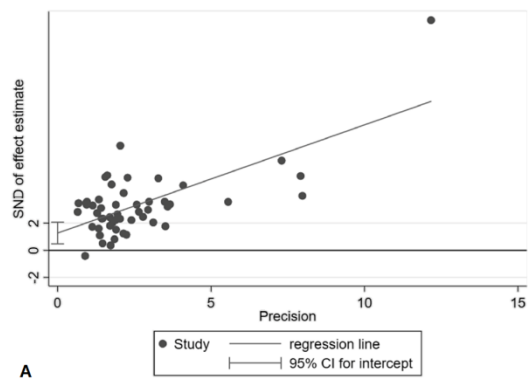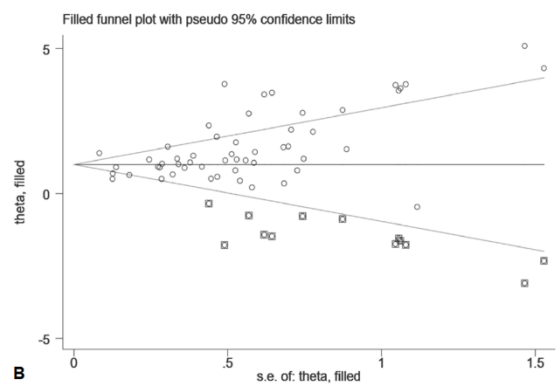

Supplement: S1 Fig — (A) Egger’s publication bias plots for the assessment of potential publication bias. Asymmetry of the dot distribution between regression lines showed potential publication bias, P = 0.002, t = 3.19, 95%CI (0.47, 2.07). (B) The funnel plot of publication bias by the trim-and-fill method. After filled 13 potentially missing studies, the funnel plots were symmetrical. CI, confidence interval. (PDF) [file pone.0248812.s003.pdf]

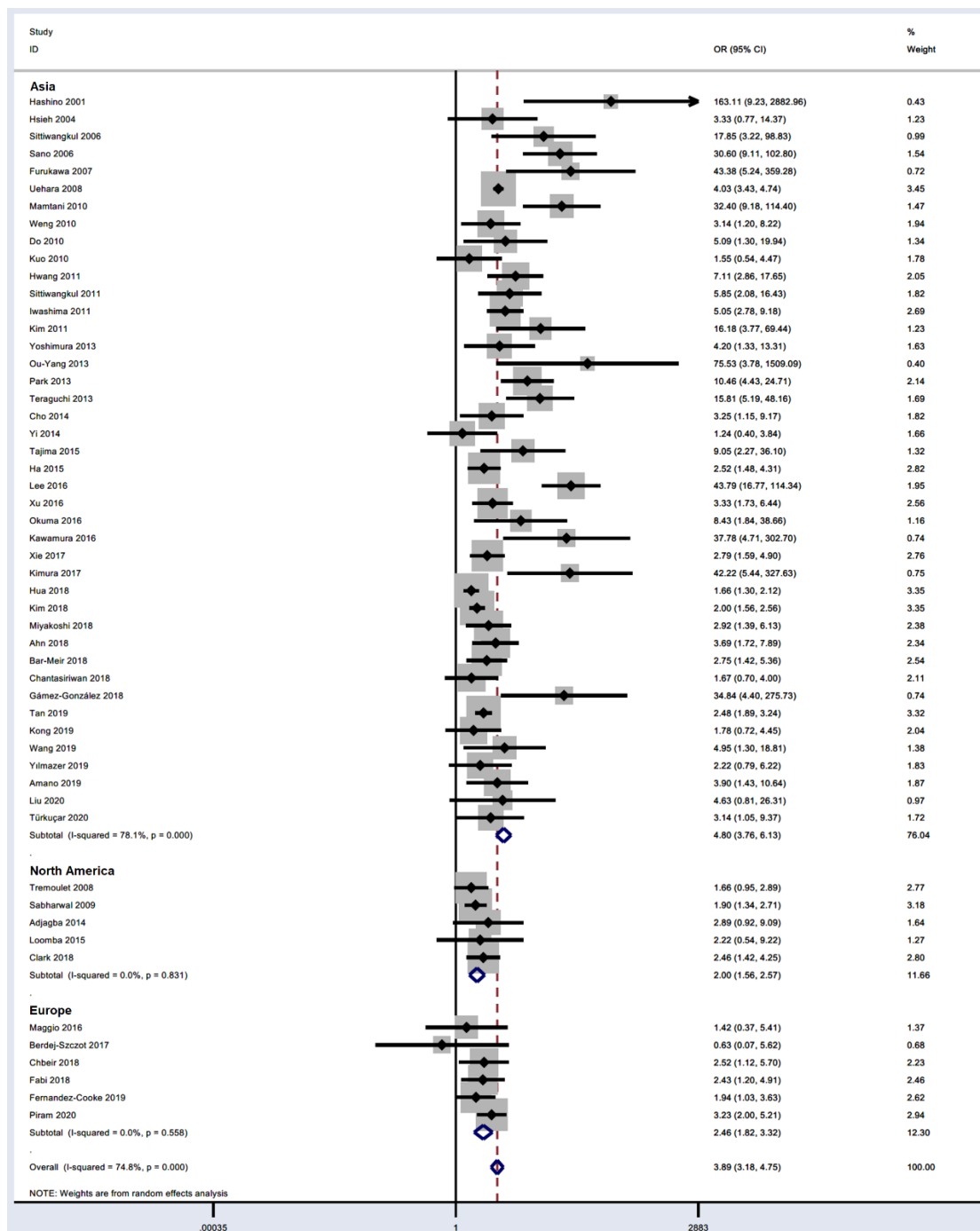

Supplement: S2 Fig — (PDF) [file pone.0248812.s004.pdf]

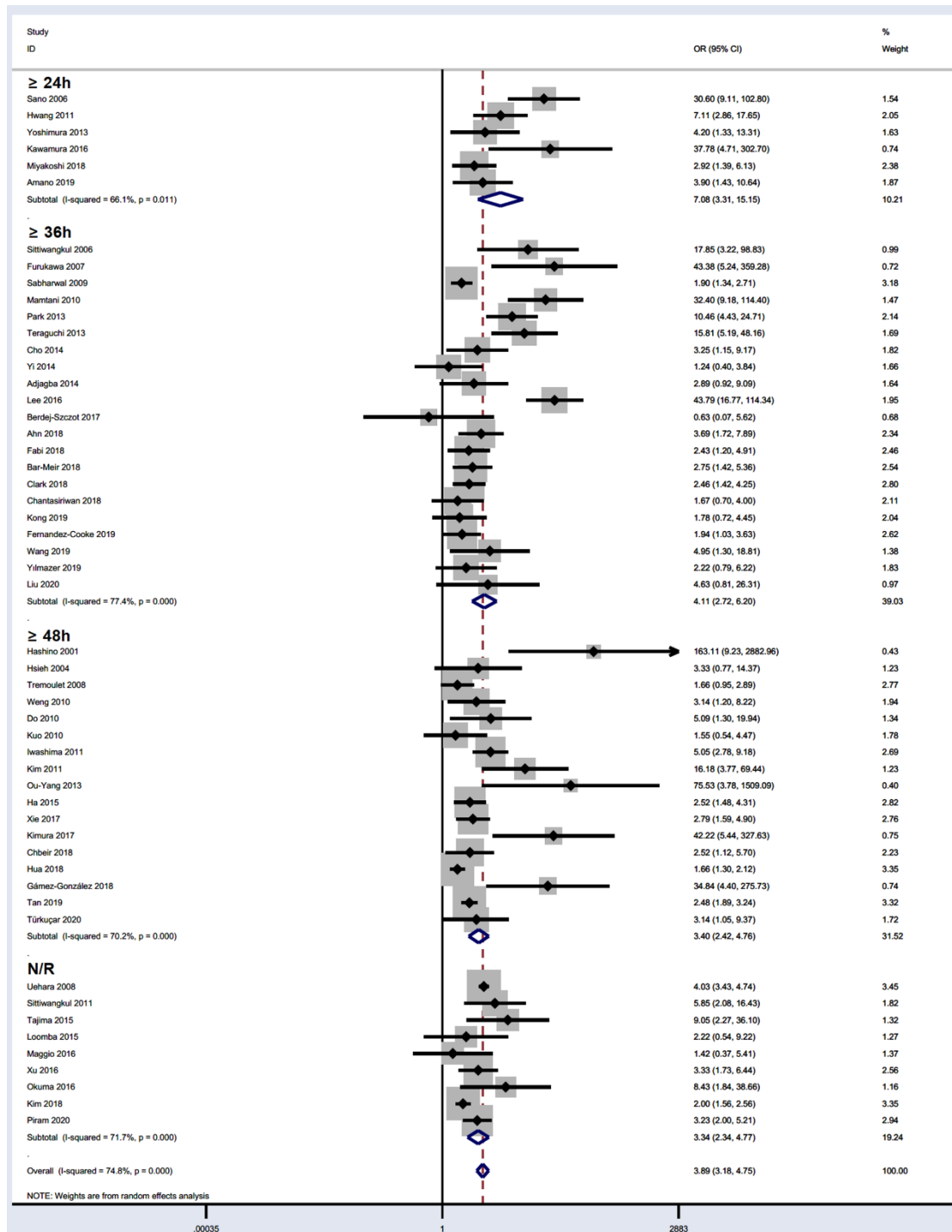

Supplement: S3 Fig — (PDF) [file pone.0248812.s005.pdf]

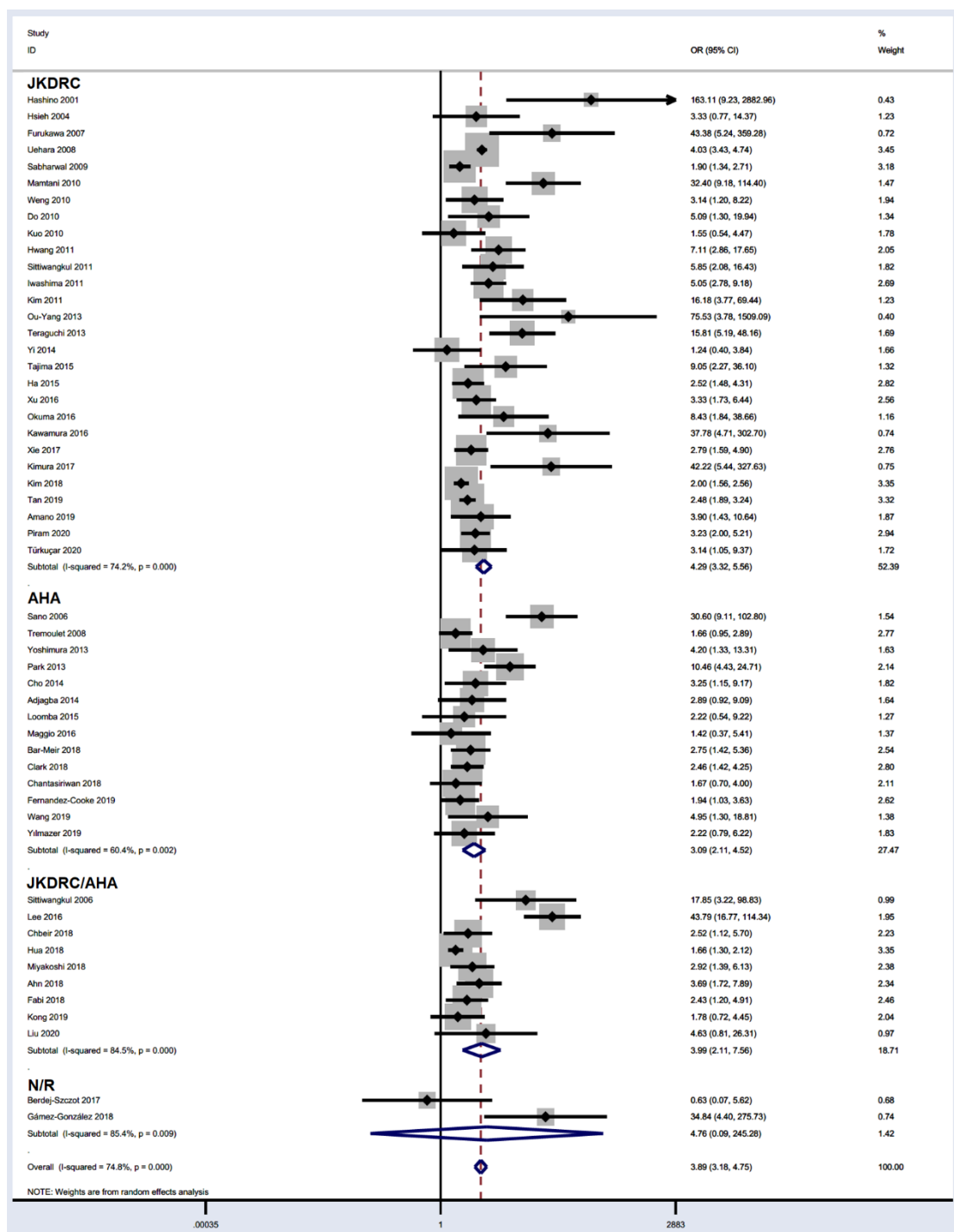

Supplement: S4 Fig — (PDF) [file pone.0248812.s006.pdf]

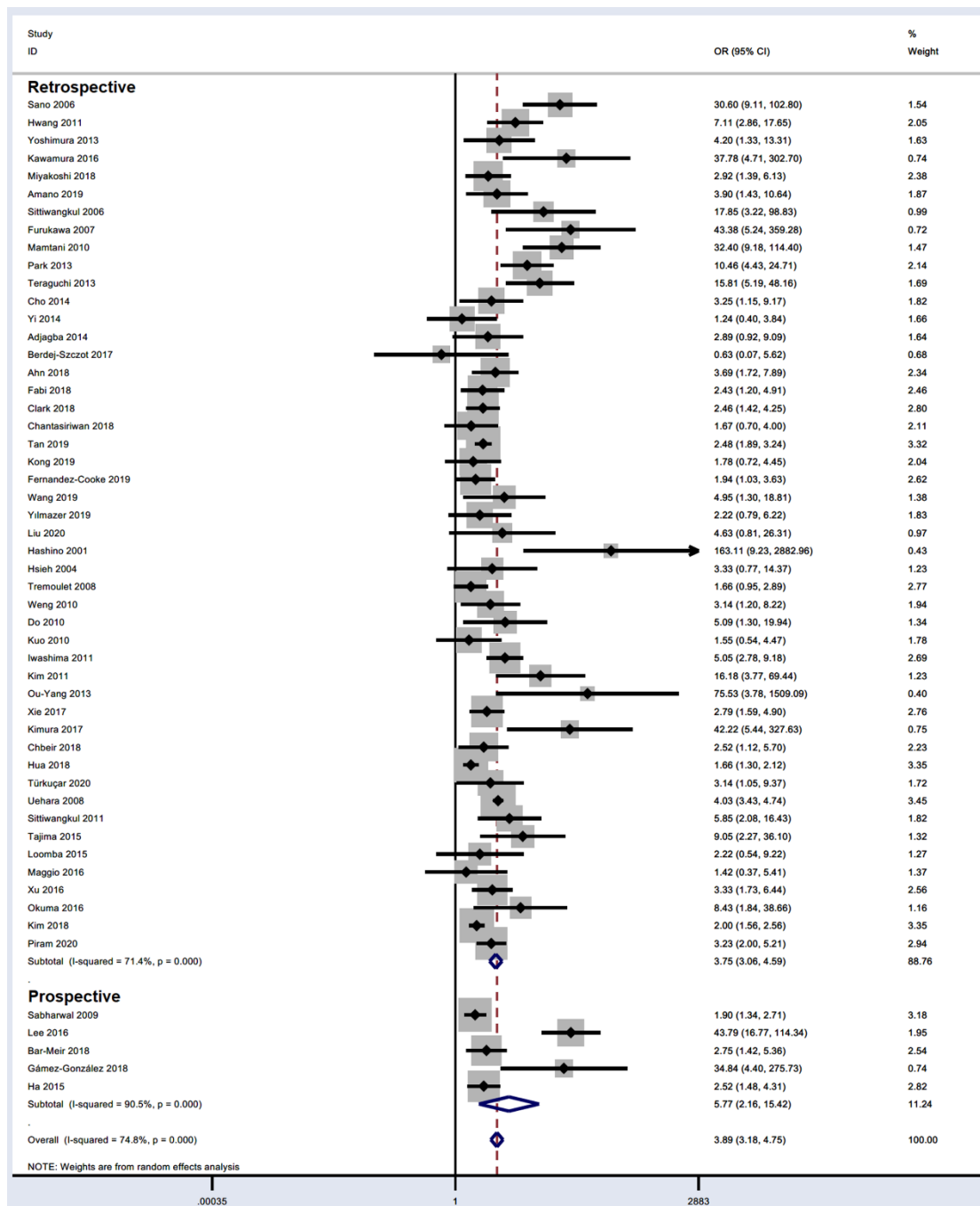

Supplement: S5 Fig — (PDF) [file pone.0248812.s007.pdf]
